# The Nucleolar Protein C1orf131 Is a Novel Gene Involved in the Progression of Lung Adenocarcinoma Cells through the AKT Signalling Pathway

**DOI:** 10.3390/ijms25126381

**Published:** 2024-06-09

**Authors:** Zhili Wei, Yiming Zhao, Jing Cai, Yajun Xie

**Affiliations:** 1 The Ministry of Education Key Laboratory of Laboratory Medical Diagnostics, the College of Laboratory Medicine, Chongqing Medical University, Chongqing 400016, China; weizhili@126.com; 2 College of Medical Informatics, Chongqing Medical University, Chongqing 400016, China; zhaoyiminglalala@163.com; 3 National Talent Introduction Demonstration Base, the College of Basic Medicine, Harbin Medical University, Harbin 150081, China; caijing@stu.cqmu.edu.cn

**Keywords:** C1orf131, nucleolar protein, lung cancer, cell cycle, proliferation

## Abstract

Lung adenocarcinoma (LUAD) is the most widespread cancer in the world, and its development is associated with complex biological mechanisms that are poorly understood. Here, we revealed a marked upregulation in the mRNA level of C1orf131 in LUAD samples compared to non-tumor tissue samples in The Cancer Genome Atlas (TCGA). Depletion of C1orf131 suppressed cell proliferation and growth, whereas it stimulated apoptosis in LUAD cells. Mechanistic investigations revealed that C1orf131 knockdown induced cell cycle dysregulation via the AKT and p53/p21 signalling pathways. Additionally, C1orf131 knockdown blocked cell migration through the modulation of epithelial–mesenchymal transition (EMT) in lung adenocarcinoma. Notably, we identified the C1orf131 protein nucleolar localization sequence, which included amino acid residues 137–142 (KKRKLT) and 240–245 (KKKRKG). Collectively, C1orf131 has potential as a novel therapeutic marker for patients in the future, as it plays a vital role in the progression of lung adenocarcinoma.

## 1. Introduction

Lung cancer accounts for the highest rates of both occurrence and mortality of any cancer worldwide [1]. Lung cancer is classified into two main groups: non-small cell lung cancer (NSCLC) and small cell lung cancer (SCLC). NSCLC, which includes lung adenocarcinoma (LUAD) and lung squamous cell carcinoma (LUSC), comprises 85% of all lung cancers, whereas SCLC comprises 15% [2]. Within the NSCLC classification, LUAD is among the most prevalent subtypes [3,4,5]. Although advancements have been made in the early detection and targeted therapy of patients diagnosed with LUAD, the overall 5-year patient survival rate remains comparatively modest at 19% [6,7,8]. In recent years, targeted therapeutic approaches that target LUAD driver genes have been shown to increase the survival rate of patients [9]. Therefore, investigating the molecular mechanisms associated with LUAD is essential for identifying potential biomarkers and developing new therapeutic strategies [10,11].

Protein synthesis in all cells is catalysed by ribosomes, which couple the decoding of messenger RNA (mRNA) from ribosomal small subunits to peptide bonds from large ribosomal subunits [12,13,14]. Human ribosome assembly involves a wide variety of assembly factors that first form an enormous precursor of the ribosomal small subunit (SSU), known as the SSU processome [14,15]. The C1orf131 gene is located on chromosome 1 in open reading frame 131, and it encodes a chromosome periphery protein that is involved in mitosis [16]. During SSU processome formation in the nucleolus, numerous ribosome biogenesis-related factors [17,18], including RNA chaperones and ribosomal proteins, bind to nascent preribosomal RNA and work together to mediate RNA folding, modification, rearrangement and cleavage [18,19]. In addition, RNA exosomes are responsible for the targeted degradation of preribosomal RNA [20]. C1orf131 prevents the recruitment of the helicase DHX37 before the post-A1 state [21,22]. Thus, recent research confirmed that the C1orf131 gene is part of the SSU processome and that it is a pre-A1 state-specific factor [12]. Indeed, few reports have demonstrated the function of C1orf131 in cancer progression. During our laboratory research on other topics related to the occurrence and development of LUAD, high-throughput sequencing analysis revealed that this gene is highly expressed in LUAD. At the same time, we validated this result in multiple clinical samples of LUAD using RT-PCR (*n* = 4), and therefore we hypothesized that this gene may play an important role in the carcinogenesis process in LUAD.

A hallmark of cancer is sustained proliferative signalling, which drives excessive rounds of cell division [23,24]. Recent insights have shown that this change is caused by mutations in proteins that inhibit apoptosis and that participate in pathways that regulate cell cycle progression rather than mechanisms that promote uncontrolled cell cycle progression [25]. The cell cycle is regulated by various signalling pathways, including pathways that involve cell cycle proteins and cyclin-dependent kinases (CDKs) [26], pathways that transcriptionally regulate G1-S progression [27,28], checkpoint signalling pathways [29] and pathways that regulate ubiquitin ligases [30]. Notably, the p53 gene is a well-known tumour suppressor that is crucial in inducing apoptosis and cell cycle arrest [31,32]. The cyclin-dependent kinase inhibitor p21 was the first transcriptional target of p53 to be identified [33,34]. After p53 activation, for example, by DNA damage induction or viral infection, p21 expression is upregulated [35]. High levels of p21 prompt the assembly of the RB-E2F complex and the downregulation of a variety of cell cycle-related genes. These changes induce G1 cell cycle arrest and prevent entry into the S phase [33,36]. Moreover, aberrant mutation and activation of the PI3K/Akt pathway enhance cell growth, motility, proliferation, and metabolism in many cancers [37].

In this study, we aimed to confirm that C1orf131 is overexpressed in various cancers, such as LUAD, liver hepatocellular carcinoma (LIHC), lymphoid neoplasm, and bladder urothelial carcinoma (BLCA) from TCGA database. Silencing C1orf131 expression inhibits cell growth and migration by reducing the levels of EMT-associated proteins while inducing cell cycle arrest. Moreover, we determined the nucleolus localization sequence in C1orf131. Our findings identified a novel molecular mechanism by which C1orf131 functions in LUAD, and these findings might provide a novel therapeutic approach to prevent LUAD proliferation and metastasis.

## 2. Results

### 2.1. C1orf131 Is Overexpressed in LUAD and Correlates with a Worse Prognosis

**Figure 1:**
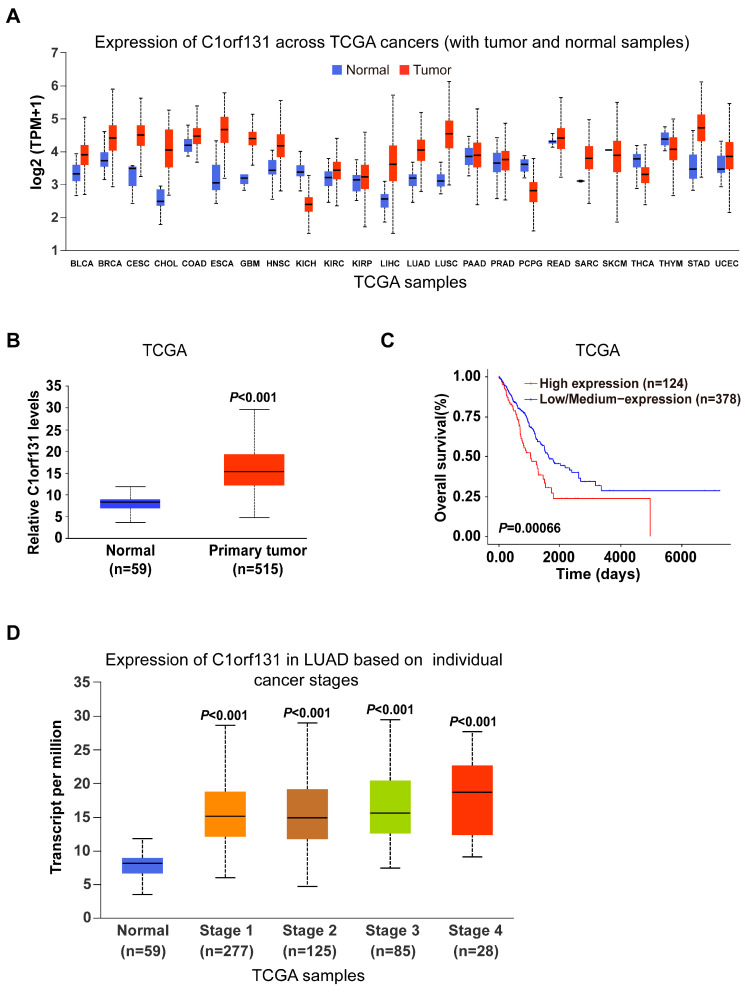
C1orf131 expression was significantly elevated in LUAD tissues. (**A**) C1orf131 mRNA levels in various tumour tissues and adjacent normal tissues based on UALCAN. (**B**) Verification of C1orf131 mRNA overexpression in 515 LUAD tissues and 59 adjacent normal tissues from TCGA. (**C**) Based on C1orf131 expression, Kaplan–Meier analysis was applied to estimate overall survival in patients with LUAD from TCGA. (**D**) The expression of C1orf131 is correlated with the clinical stage of LUAD from the TCGA.

To evaluate the expression patterns of C1orf131 in various malignancies, we performed analyses through the University of Alabama at the Birmingham Cancer Data Analysis Portal (UALCAN). We found that, in comparison to that in normal tissues, C1orf131 expression was elevated in LIHC, BLCA, CECS, and LUAD tissues. However, C1orf131 was expressed at low levels in pheochromocytoma (PCPG), kidney chromophobe (KICH) and thyroid carcinoma (THCA) (Figure 1A). RNA sequencing data, which included 515 lung adenocarcinoma tissues and 59 adjacent normal tissues, were accessed from the TCGA database. C1orf131 expression was markedly elevated in the tissues of LUAD patients (Figure 1B). Kaplan–Meier survival analyses were conducted with the online bioinformatics tool UALCAN, and the results illustrated that elevated C1orf131 expression was linked to worse overall survival in LUAD (Figure 1C). In addition, the expression of the C1orf131 gene was strongly associated with the clinical stage of LUAD (Figure 1D). Collectively, these results demonstrated that C1orf131 was overexpressed in LUAD patients with a poor prognosis. C1orf131 could serve as a novel biomarker for LUAD.

### 2.2. Silencing C1orf131 Significantly Inhibits Cell Proliferation in Lung Cancer

**Figure 2:**
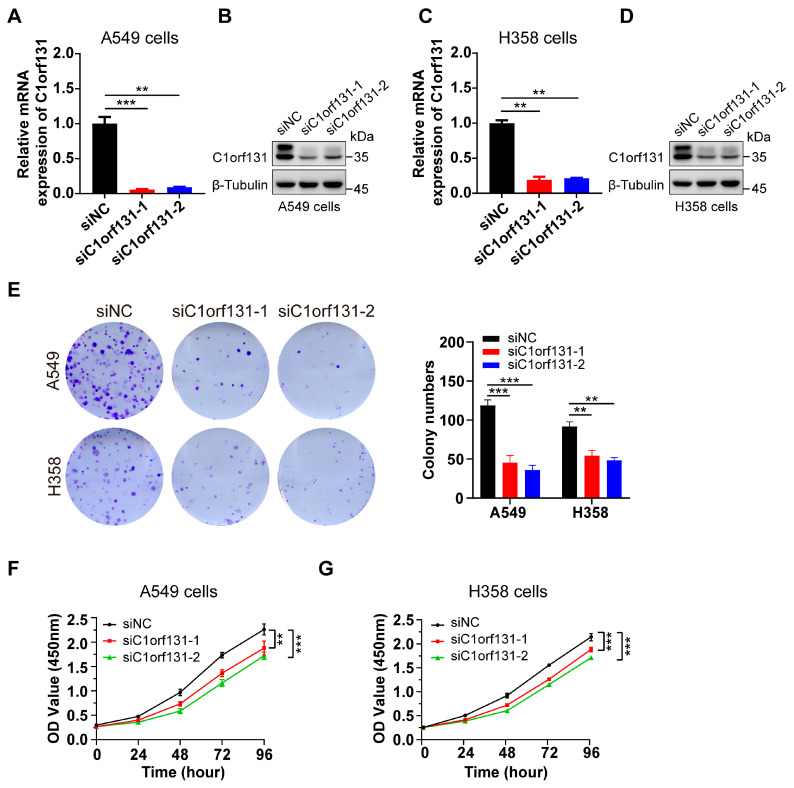
C1orf131 knockdown suppresses LUAD cell proliferation. (**A**,**B**) siRNA-mediated C1orf131 gene knockdown. The control siRNA (siNC) or siRNA targeting C1orf131 (siC1orf131-1 or siC1orf131-2) was transiently transfected into human A549 cells. Total RNA from the cell and cell lysates was subjected to RT–PCR and Western blotting, respectively, 48 h after transfection. (**C**,**D**) C1orf131 expression levels in transfected H358 cells were examined via RT–PCR and immunoblotting. (**E**) Colony formation assays were performed in the siNC and siC1orf131 groups. After the cells were cultured for approximately 12 days, the number of clones generated by each group of cells was counted independently. (**F**,**G**) The CCK-8 assay was used to evaluate cell viability in two groups (siNC and siC1orf131). All the experiments were independently conducted three times. ** *p* < 0.01; *** *p* < 0.001; ns, not significant.

To elucidate the effect of C1orf131 on the pathogenesis of LUAD, we examined the effect of siRNA-mediated C1orf131 knockdown on the growth of two different human LUAD cell lines, namely, the A549 and H358 cell lines. Both RT–PCR and immunoblotting analyses confirmed that the C1orf131 level was significantly reduced, while the expression of the internal control genes β-Tubulin remained unaltered (Figure 2A–D). Colony formation assays also revealed that silencing C1orf131 significantly lowered the number of colonies (Figure 2E). Furthermore, the CCK-8 results suggested that C1orf131 knockdown greatly attenuated the proliferation of A549 and H358 cells (Figure 2F,G). These findings indicate that C1orf131 knockdown prevents the growth of LUAD cells.

### 2.3. C1orf131 Affects Cell Cycle Distribution and the Apoptosis of LUAD Cells

**Figure 3:**
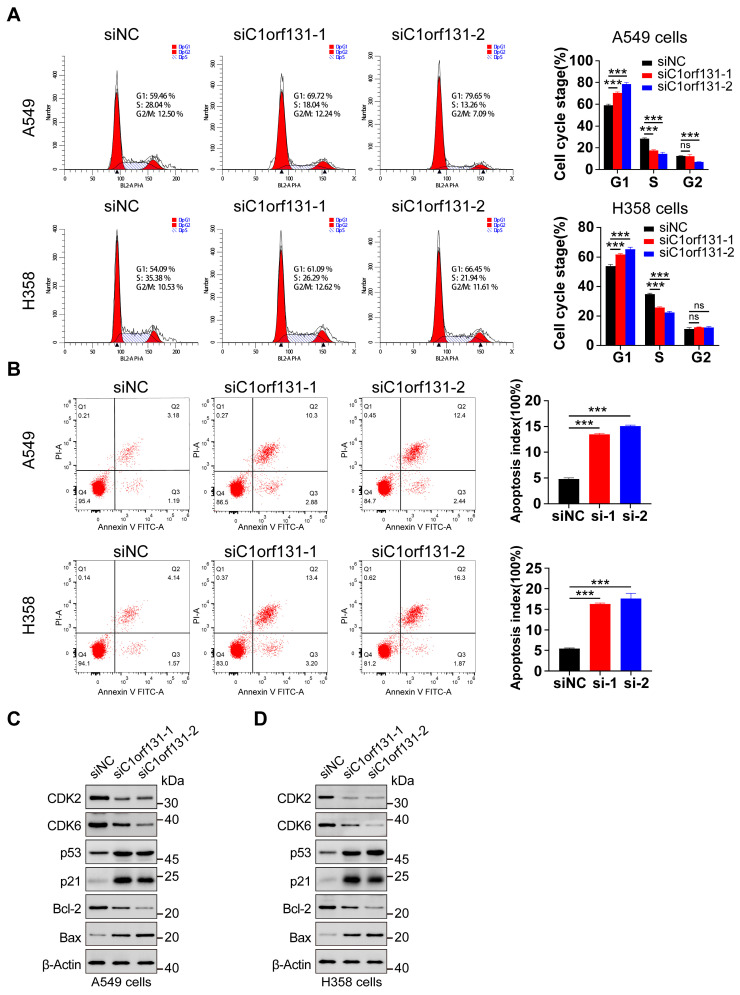
Silencing C1orf131 affects the cell cycle arrest and apoptosis of LUAD cells. A549 and H358 cells were transfected with siNC or siC1orf131, and then cell cycle analysis (**A**) and apoptosis assays (**B**) were performed. (**C**,**D**) The expression of the cell cycle-associated proteins CDK2, CDK6, p53 and p21, as well as the apoptosis-related proteins Bcl-2 and Bax, were analysed using Western blotting in A549 and H358 cells. The data are presented as the mean ± SD. All the experiments were independently conducted three times. *** *p* < 0.001; ns, not significant.

To determine whether C1orf131 knockdown influences cell cycle progression, we used flow cytometry. As shown in Figure 3A, silencing C1orf131 caused significant cell cycle arrest of A549 and H358 cells in the S phase. The percentages of siRNA-transfected A549 cells arrested in the S phase were 18.04% and 13.26%, respectively, whereas the proportion of arrested cells in the siRNA control group accounted for 28.04%, as indicated by the decreased percentages of cells in the S phase. Similar results were obtained in H358 cells (Figure 3A). Notably, G1/S-phase-related checkpoint proteins such as CDK2 and CDK6 were markedly downregulated. In addition, C1orf131 knockdown increased p53 and p21 expression in A549 and H358 cells, which are responsible for cell cycle regulation. (Figure 3C,D).

To further determine whether C1orf131 knockdown triggers cell apoptosis, we used Annexin V-FITC and PI double staining and flow cytometry to detect apoptosis. At 36–48 h after transfection, 13.18% and 14.84% of the C1orf131-knockdown A549 cells were in the early and late apoptotic stages, respectively. But only 4.37% of the control cells were apoptotic. Similarly, C1orf131 knockdown in H358 cells also increased the percentage of apoptotic cells with approximately 16.6% and 18.17% of cells in the early and late apoptotic stages, respectively, compared to 5.71% of control cells (Figure 3B). The expression of the apoptosis-related protein Bax was upregulated, whereas that of the anti-apoptotic protein Bcl-2 was reduced in C1orf131-knockdown in A549 and H358 cells (Figure 3C,D).

### 2.4. C1orf131 Silencing Suppresses the Migration of LUAD Cells

**Figure 4:**
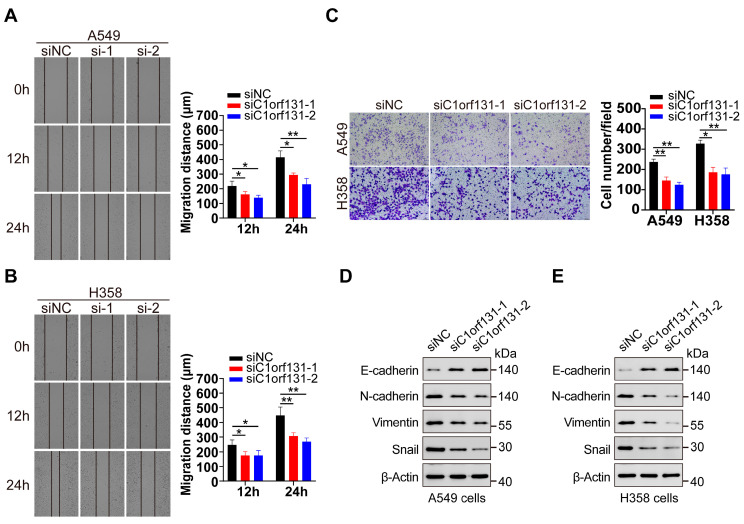
Silencing C1orf131 inhibits migration of LUAD cells. (**A**,**B**) Wound healing assays were performed in C1orf131-knockdown A549 and H358 cells. (**C**) C1orf131-knockdown cells were subjected to Transwell assays to assess cell migration. Representative images (40×) and quantification results are presented for each assay. (**D**,**E**) The expression of EMT-associated proteins in the siNC and siC1orf131 groups was assessed by immunoblotting in A549 and H358 cells. The data are presented as the mean ± SD. All the experiments were independently conducted three times. * *p* < 0.05; ** *p* < 0.01; ns, not significant.

We further explored whether C1orf131 regulates LUAD cell migration in vitro. The wound healing assays revealed that C1orf131 knockdown obviously slowed the lateral migration rate of cells towards the wound in a confluent cell monolayer (Figure 4A,B). Moreover, the Transwell results showed that the migration ability of H358 and A549 cells was attenuated when C1orf131 was knocked down (Figure 4C). Given the close association between EMT and tumour cell migration [38,39], our results revealed that C1orf131 knockdown caused an elevation of E-cadherin and a decline in N-cadherin, Vimentin, and Snail expression in A549 and H358 cells (Figure 4D,E). These findings collectively suggested that C1orf131 might regulate the migration of LUAD cells by modulating the EMT.

### 2.5. Knockdown of C1orf131 Decreases LUAD Cell Viability In Vivo

**Figure 5:**
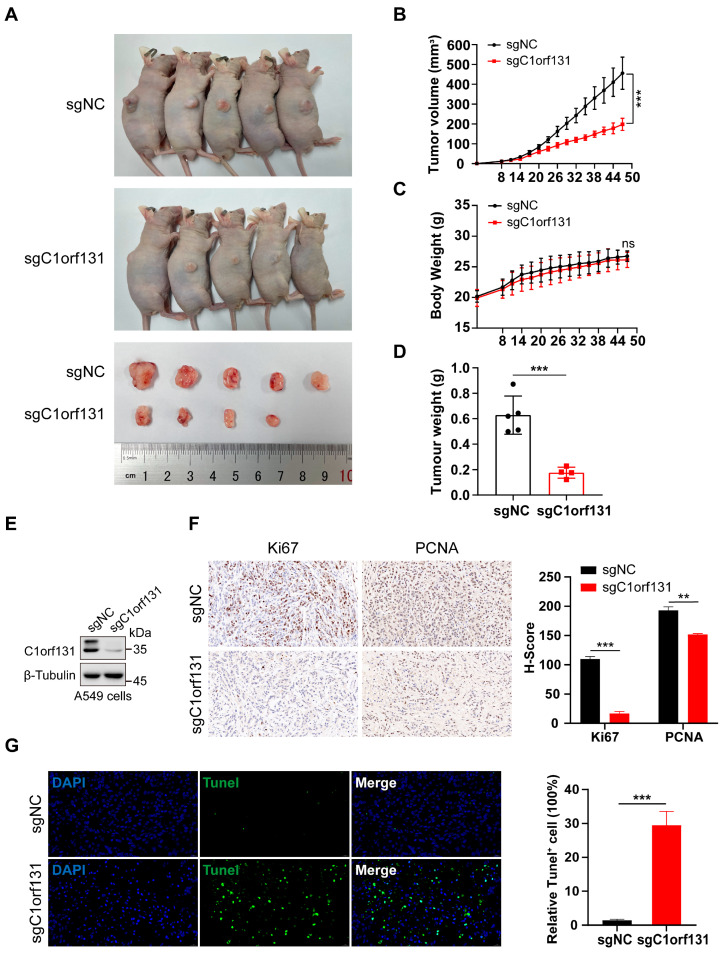
C1orf131 contributes to LUAD growth in vivo. (**A**) After 47 days, subcutaneous tumours were observed in mice that were injected with A549-sgNC or A549-sgC1orf131 cells. Tumour volume (**B**), body weight (**C**) and tumour weight (**D**) of the xenografts from the A549-sgNC and A549-sgC1orf131 groups over 47 days are shown. (**E**) Immunoblotting was analysed to measure the knockdown efficiency with stable C1orf131 knockout A549 cells administered to mice by subcutaneous injection. (**F**,**G**) Representative images of IHC staining (40×) and TUNEL staining (63×) were obtained from tumours harvested from xenograft model mice. ** *p* <0.01; *** *p* <0.001; ns, not significant. Representative images are shown with a scale bar of 20 μm.

C1orf131-knockout A549 cells were generated via lentivirus-mediated CRISPR/Cas9 technology [40,41], and immunoblotting analysis revealed that C1orf131 was stably knocked out at the protein level (Figure 5E). Furthermore, animal experiments were established to explore the effects of C1orf131 on the proliferation ability of cancer cells, initially using a subcutaneous tumour implantation model in BALB/c nude mice. During the 47-day observation period, mice that were implanted with A549-sgC1orf131 knockout cells exhibited slower tumour growth and a corresponding decrease in average tumour weight compared to those of mice bearing control tumours (Figure 5A–D). However, there was no significant difference in body weight between the sgNC and sgC1orf131 groups of mice (Figure 5C). IHC staining revealed decreased expression of the proliferation markers Ki67 and PCNA in the A549-sgC1orf131 groups (Figure 5F). Additionally, TUNEL staining indicated that A549-sgC1orf131 xenografts exhibited greater apoptosis than the A549-sgNC (Figure 5G).

### 2.6. The Nucleolus Localization Sequence of C1orf131 Includes Amino Acids 137–142 and 240–245

**Figure 6:**
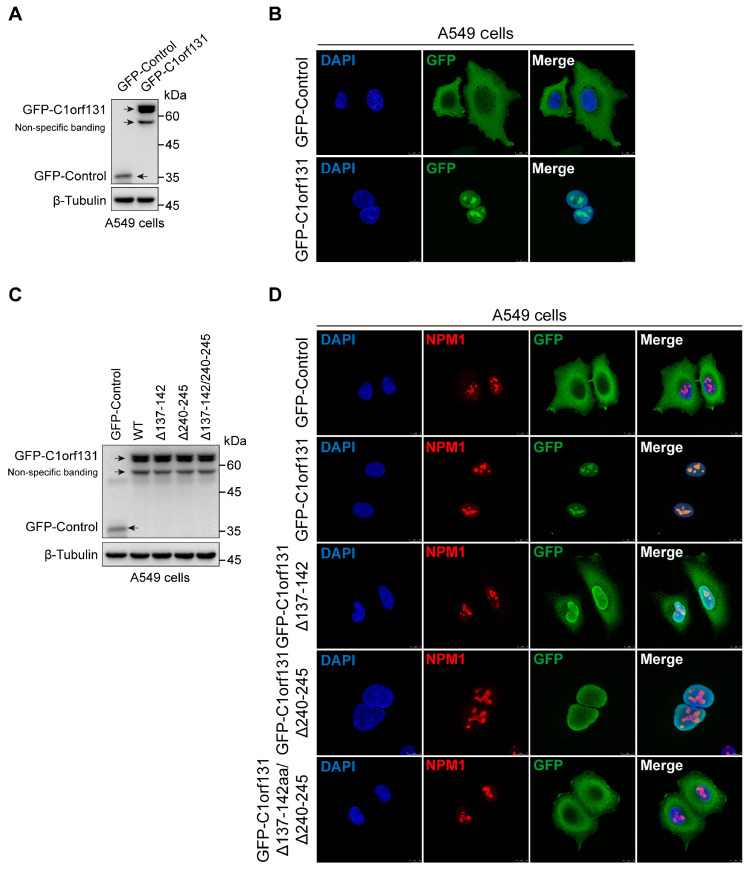
The NLS of C1orf131 includes amino acids 137–142 and 240–245. (**A**) A549 cells stably overexpressing GFP-Control or GFP-C1orf131 were generated. The efficiency of GFP-Control and GFP-C1orf131 expression was determined by immunoblotting. (**B**) The cells were subjected to immunofluorescence staining with an anti-GFP antibody, the corresponding secondary antibody and DAPI in GFP-Control or GFP-C1orf131 group. (**C**) Immunoblotting was performed to measure the expression of truncated C1orf131 mutants in A549 cells. (**D**) Immunofluorescence staining was performed to determine the subcellular distribution of GFP-Control, GFP-C1orf131, and truncated C1orf131 mutants. Representative images are shown with a scale bar of 10 μm.

To probe the biological function of C1orf131, we determined the subcellular location of C1orf131 by immunofluorescence. The results revealed that in A549 cells, C1orf131 was located mainly in the nucleolus (Figure 6A,B). We predicted that the nuclear localization sequences (NLSs) of C1orf131 were amino acids 137–142 (KKRKLT) and amino acids 240–245 (KKKRKG) by using an online website (https://rostlab.org/services/nlsdb/, accessed on 25 May 2023.). We constructed different truncated mutants of C1orf131 by deleting the NLS, including Δ137–142 (deletion of amino acids 137–142), Δ240–245 (deletion of amino acids 240–245), and Δ137–142/Δ240–245 (deletion of amino acids 137–142 and 240–245).

We overexpressed wild-type C1orf131 and the C1orf131 truncation mutants in A549 cells (Figure 6C). However, immunofluorescence analyses revealed that the Δ137–142 mutant lacked the NLS of C1orf131, and most of this protein was located in the nucleus, while a small portion was present in the cytoplasm. In contrast, the Δ240–245 mutant remained in the nucleus, although the NLS of C1orf131 was absent. Notably, the C1orf131 mutant lacking both the 137–142 and 240–245 amino acid sequences (Δ137–142/Δ240–245) was located in the cytoplasm, and its ability to localize to the nucleolus was completely lost (Figure 6D). Taken together, these data indicated that amino acids 137–142 and 240–245 of the NLS were involved in the specific subcellular localization of C1orf131. Deletion of these sequences from wild-type C1orf131 resulted in the translocation of C1orf131 from the nucleolus to the cytoplasm.

### 2.7. Identification of Oncogenic Signatures and Signalling Pathways That Were Correlated with C1orf131

**Figure 7:**
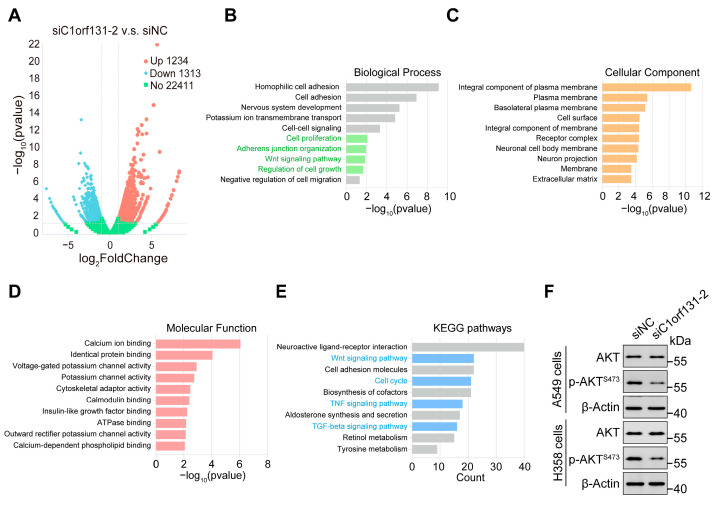
Identification of downstream genes and signalling pathways that are modulated by C1orf131. (**A**) RNA-seq analyses were conducted with negative control and C1orf131-knockdown A549 cells. The volcano plot displays the DEGs. (**B**–**E**) GO and KEGG pathway enrichment analyses were performed on the 2547 DEGs between the siNC and siC1orf131-2 groups. (**F**) The expression of key proteins associated with cell proliferation-related pathways was measured by immunoblotting.

To further explore the potential molecular mechanisms underlying C1orf131-associated lung cancer carcinogenesis, we conducted RNA-seq analyses to determine changes in the gene expression profiles of C1orf131-knockdown cells at the genome-wide level. We identified 2547 differentially expressed genes (DEGs) upon C1orf131 knockdown, of which 1313 were downregulated and 1234 were upregulated (Figure 7A). Gene Ontology (GO) analysis found that the DEGs were mainly abundant in cell proliferation, the Wnt signalling pathway, regulation of cell growth, adherens junction organization, etc. (Figure 7B–D). KEGG analysis indicated that the DEGs were implicated in tyrosine metabolism, cell cycle, Wnt signalling, TNF signalling and TGF-beta signalling pathways (Figure 7E). Moreover, Western blotting verified that knockdown of C1orf131 caused a dramatic decline in the level of phosphorylated Akt at serine 473 (*p*-Akt ^S473^) without affecting total Akt protein levels (Figure 7F). This observation suggested that C1orf131 has a role in stimulating cell proliferation and migration through the Akt pathway in LUAD. Collectively, these studies imply that C1orf131 may act as a critical regulator of certain tumour-related pathways in LUAD.

## 3. Discussion

Previous studies have used multiclassifier combinatorial proteomics and machine learning to reveal functional connections between protein complexes in mitotic chromosomes. These studies identified approximately 560 proteins that were previously uncharacterized, and these findings will facilitate further research. C1orf131, also known as cPERP-A, is a chromosome periphery protein that is involved in mitosis. The function of the chromosome periphery, which is enriched with nucleolar hitchhiker proteins and ribosomes, is not known [42]. Several researchers have reported that C1orf131 is an RNA-binding protein (RBP) that exhibits mRNA-binding activity in human HeLa cells [43,44]. However, the potential role and molecular mechanisms of the C1orf131 gene in tumorigenesis and development have not yet been investigated. In this study, we initially analysed C1orf131 mRNA expression in various human tumour samples from the TCGA. The expression of C1orf131 was significantly elevated in CECS, LIHC, and LUAD tissues compared with adjacent normal tissues. However, kidney chromophobe, pheochromocytoma, paraganglioma, thyroid carcinoma and thymoma exhibit low levels of C1orf131 expression. Notably, our functional experiments confirmed that C1orf131 has a vital function in the regulation of cell proliferation both in vivo and in vitro. Knockdown of C1orf131 markedly delayed cell cycle progression. Moreover, C1orf131 knockdown significantly reduced A549 and H358 cell migration, as demonstrated by both wound healing and Transwell assays. This study provides the first evidence that C1orf131 is a nucleolar protein that promotes the proliferation and migration of tumour cells in LUAD.

Nucleophosmin (NPM1) is an abundant multifunctional nucleolar protein that is ubiquitously expressed in mammalian cells [45,46]. NPM1 is primarily involved in the maintenance of ribosome biogenesis and genome stability, and it can affect a variety of biological processes [47]. It has been shown that NPM1 interacts with the oncogenic transcription factor FOXM1 in cancer cells. Interestingly, NPM1 knockdown attenuated the proliferation and growth of pancreatic adenocarcinoma cells. In addition, NPM1 was also found to be overexpressed in colon, breast, gastric, thyroid and prostate tumours [48,49,50]. In this study, we found that C1orf131 is a nuclear protein with two nucleolar localization sequences, including amino acids 137–142 (KKRKLT) and 240–245 (KKKRKG). Immunofluorescence experiments confirmed the cellular colocalization of NPM1 and C1orf131 in A549 cells, but the specific mechanism that regulates this association remains to be elucidated. Recent studies have reported that NPM1 is also overexpressed in LUAD and is significantly correlated with clinical stage and prognosis [51,52]. However, the biological functions of the C1orf131 protein nucleolar localization sequence in LUAD are currently unknown.

EMT is a reversible cellular event in which epithelial cells lose polarity and adhesion after undergoing molecular reprogramming and phenotypic alterations to transiently acquire a mesenchymal stem cell-like phenotype [53]. Aberrant mutation and activation of the PI3K/Akt pathway enhance cell growth, motility, proliferation and metabolism in many human cancers [37]. Western blotting confirmed that knockdown of C1orf131 dramatically decreased the Akt phosphorylation at serine 473 without affecting total Akt protein levels. C1orf131 played a role in promoting cell migration through the Akt pathway. Additionally, the Akt signalling pathway affects the expression of several downstream molecules, including Bcl-2, Bax, p53, and cyclin D1, which are involved in cell proliferation, anti-apoptotic mechanisms, and cell cycle arrest. Activation of the PI3K/Akt pathway is one of the drivers of tumour metastasis and invasion [37]. Dysfunction of components of the PI3K/AKT signalling pathway, such as the loss of PTEN function and activation of AKT, are well-known drivers of resistance to anticancer therapy. Many inhibitors directed against the PI3K/Akt signalling pathway have been developed, all of which are attractive strategies for tumour therapy [54].

Based on our findings and reports on C1orf131, we have established a correlation between the expression of C1orf131 and the progression of LUAD. Furthermore, we have elucidated the specific motif on C1orf131 that enables its localization within the nuclear compartment. Additionally, our results suggest that the involvement of C1orf131 in regulating EMT, cell cycle, and apoptosis may be a principal factor contributing to its role in the progression of LUAD. Although we have successfully confirmed the nucleolar localization of C1orf131, the specific functions and signalling pathways associated with its nuclear presence remain unknown. Our future investigations will focus on exploring the nuclear role of C1orf131 in detail, including its relevance and clinical implications in various malignant tumours and related diseases using both publicly available databases and experimental methods. Further studies are essential to understand the impact of deleting the nucleolar localization sequence of C1orf131 on the malignant phenotype of LUAD, both in vivo and in vitro. Notably, we have not yet determined whether there exists a protein interaction between C1orf131 and NPM1, nor have we explored the effects of manipulating C1orf131 expression levels on the localization of NPM1 within nucleoli. Future experiments are essential to explore potential regulatory molecules that might work in conjunction with C1orf131 to impact the malignant progression of LUAD. Furthermore, the association of C1orf131 with the classical PI3/AKT pathway remains to be investigated.

In conclusion, our data indicate that C1orf131 is overexpressed in LUAD cells and participates in cell growth, motility and cell cycle progression upon the p53/p21 and AKT signalling pathways. In addition, we showed that C1orf131 is a nucleolar protein that is essential for maintaining the malignant phenotype of LUAD cells. Therefore, C1orf131 might be a promising biomarker for personalized diagnosis and therapy of lung adenocarcinoma.

## 4. Materials and Methods

### 4.1. Cell Culture

This study utilized two human LUAD cell lines, A549 and H358, which were purchased by the Meisen CTCC Company (Hangzhou, China). Cell culture is typically performed in DMEM/F12 containing 10% dialyzed foetal bovine serum (FBS, Excell, Suzhou, China) and penicillin–streptomycin (HyClone, Beijing, China). Cells were cultivated in a 37 °C, 5% CO_2_ incubator. The cell lines were authenticated via the cell library from which they were obtained and were not contaminated with mycoplasma.

### 4.2. Small Interfering RNA (siRNA)-Mediated Knockdown

C1orf131-specific siRNA nucleotide sequences and control siRNA were chemically synthesized by Beijing Tsingke Biotech Co., Ltd (Beijing, China). The indicated siRNAs were transiently transfected into LUAD cells using Lipofectamine 2000 reagent (Invitrogen, Carlsbad, California, USA) as described in the protocol. C1orf131-siRNA1 sense (5′-3′): GGUUUGGUAUCACGGGUUA. C1orf131-siRNA1 anti-sense (5′-3′): UAACCCGUGAUACCAAACC. C1orf131-siRNA2 sense (5′-3′): CGACUUUGGAGGUACAGAA. C1orf131-siRNA2 anti-sense (5′-3′): UUCUGUACCUCCAAAGUCG. Control siRNA sense (5′-3′): UUCUCCGAACGUGUCACGU. Control siRNA anti-sense (5′-3′): ACGUGACACGUUCGGAGAA.

### 4.3. Lentivirus-Mediated C1orf131 Overexpression in Cells

The full-length or truncated sequence of the human C1orf131 gene was inserted into the lentiviral-mediated plasmid LV-CMV-CTL with an N-terminal GFP tag. The accuracy of the resulting sequences was checked by sequencing after successful vector construction, and these vectors were named WT C1orf131 (GFP-C1orf131), Δ137–142 (deletion of amino acids 137–142), Δ240–245 (deletion of amino acids 240–245) and Δ137–142/Δ240–245 (deletion of amino acids 137–142 and 240–245). The primer sequences utilized to construct the plasmids are shown in Table S1. Cells were seeded at 60–70% confluence to provide optimal conditions for viral transduction. The lentiviral vectors psPAX2 and pMD2.G vectors (GeneChem, Shanghai, China) were cotransfected into HEK 293T cells. Subsequently, lentiviral solutions were obtained by collecting and concentrating the supernatant after 48 h of cell culture. After 48 h of lentiviral infection, cell lines with stable expression were selected using 2 μg/mL puromycin (Solarbio, Beijing, China).

### 4.4. Establishment of Stable C1orf131-Knockout Cells

The target sgRNAs (Table S1) were cloned and inserted into lentiCRISPR v2-dCas9 (Plasmid #112233, Addgene, Massachusetts, USA). Targeted plasmids, psPAX2 and pMD2.G vectors (Genechem, Shanghai, China) were simultaneously transfected in HEK 293T cells. After lentiviral transfection of A549 cells, the cells were selected with puromycin.

### 4.5. RNA Extraction and Quantitative Real-Time PCR (RT–PCR)

RNA was purified from cells by using RNA simple total RNA (Tiangen, Beijing, China). PrimeScript^TM^ RT Master Mix (Takara, Beijing, China) was applied to reverse transcribe total RNA into cDNA. The RT–PCR fluorescent dye utilized was 2 × SYBR Green qPCR Master Mix (Selleck, Houston, USA). In this study, the 2−ΔΔCt method was employed to calculate the relative change in the expression of the target gene, which was normalized to the mRNA level of β-Actin. First, we need to calculate ΔCt, which is the difference between the Ct value of the target gene and the Ct value of a reference gene (β-actin). ΔΔCt is the difference in ΔCt values between a test sample and a calibrator or control sample. Finally, the 2−ΔΔCt formula converts the ΔΔCt value to a fold change in relative expression [55]. The amplification sequences of primers used are summarized in Table S1.

### 4.6. Immunoblotting Analysis

Total cellular protein was harvested using 1% SDS protein lysis buffer (Beyotime, Shanghai, China) complemented with a protease inhibitor (APE × BIO, Houston, USA). The BCA assay kit was utilized to quantify the concentration of protein (Thermo Scientific, Carlsbad, California, USA). SDS-PAGE was performed to separate the proteins. Enhanced chemiluminescence solution was applied to visualize the protein bands on PVDF membranes (ECL, Smart Life Sciences, Changzhou, China). The antibodies mentioned are summarized in Table S2.

### 4.7. Cell Counting Kit-8 (CCK-8) and Colony Formation Assays

Cell proliferation and toxicity were monitored by a CCK-8 assay kit (MCE, New Jersey, USA). We added 100 µL of CCK-8 working solution to every well in 96-well plates. The cells were incubated at 37 °C for a period of 1 h, and the absorbance at 450 nm (OD 450) was read on the Thermo Scientific Microplate Reader. This procedure was repeated every 24 h. The colony formation experiments were conducted in 6-well plates, with 400 cells/well. Upon 12 days, the cells were dyed with 0.1% crystal violet (Solarbio, Beijing, China). A microscope was used to determine the numbers of colonies with over 50 cells.

### 4.8. Cell Cycle Analysis and Cell Apoptosis Assay

The cell precipitate was collected and washed three times with PBS. The cells were fixed in 75% ethanol, and subsequently analysed using a Cell Cycle Analysis Kit (MCE, New Jersey, USA). For the apoptotic studies, the cells were labelled via an Annexin V-fluorescein isothiocyanate (FITC) apoptosis assay kit (Solarbio, Beijing, China). The stained cells were finally subjected to flow cytometry analysis.

### 4.9. Wound Healing Assay

A total of 4 × 10^5^ cells were cultured overnight per well. Scratches were created on the adherent cells with the tip of a 10 μL sterile pipette gun to induce trauma. The cells were cultured in DMEM/F12 without 10% FBS. The migration of cells into the wounded area and the recovery process were monitored for 12 h and 24 h using inverted microscopy, and the entire process was documented by capturing digital images.

### 4.10. Cell Migration Assay

The Transwell culture chamber was positioned in a 24-well plate, with the Transwell chamber as the upper chamber and the 24-well plate as the lower layer (Corning, New York, USA). The upper chamber was inoculated with 2 × 10^4^ cells in DMEM/F12 without FBS, and 600 μL of DMEM/F12 containing 20% FBS was placed in the lower chamber. After 24–48 h of growth, the cells adhering to the upper surface of the filter membrane were brushed off after the cell culture medium was discarded. After fixation with 4% PFA for 20 min, the cells were washed three times in PBS and then dyed with 0.1% crystal violet for 60 min. Finally, photographic observations were made using an inverted microscope.

### 4.11. Tumour Xenograft Model

Male BALB/c nude mice were acquired from ENSIWEIER in Chongqing. They were 5–6 weeks old and weighed approximately 18–22 g. During the experiment, all mice were kept in a specific pathogen-free environment (23 ± 2 °C, 55 ± 5% relative humidity, and a 12 h light/dark cycle). They were given unrestricted access to sterilized chow with well-defined nutritional components and sterilized fresh water. A total of 10 mice were randomly divided into a control group (sgNC), an experimental group (sgC1orf131), *n* = 5 mice in each group. Each nude mouse was injected subcutaneously with 100 uL of cell suspension containing 1 × 10^6^ cells and PBS in the right axilla. Body weight and tumour volume were checked every 3 days. The formula used to calculate the tumour volume was L × W^2/2, in which the length is L and the width is W. After 47 days, a total of 10 mice were sacrificed and tumour tissues were collected and weighed. All experimental animal protocols were approved by the Ethics Committee of Chongqing Medical University, China (approval no. IACUC-CQMU-2024-0102).

### 4.12. Immunohistochemistry (IHC) Staining

Tumour tissue samples from the xenograft model were processed for paraffin embedding after fixation with 4% PFA and embedded in paraffin. IHC staining was conducted with an Elivision^TM^ Plus Polyer HRP immunohistochemistry kit (Solarbio, Beijing, China). The paraffin-embedded serial sections were 4 μm thick, and the sections were exposed to antibodies against Ki67 (1:500, ab16667) and PCNA (1:200, ab92552) at 4 °C. The samples were incubated for 2 h at room temperature with the appropriate secondary antibodies.

### 4.13. Immunofluorescence Staining

Cells were plated at appropriate densities overnight in a 37 °C incubator, fixed with 4% paraformaldehyde (PFA) for 20 min and rinsed three times in PBST. The cells were then permeabilized with 0.1% Triton X-100 for 10 min. Next, 5% bovine serum albumin was used to block cells for 120 min in a 37 °C incubator without CO_2_. After overnight incubation at 4 °C with the specified primary antibodies, the cells were subsequently treated with Alexa 488/594-conjugated secondary antibodies (Table S2) and DAPI (Solarbio, Beijing, China) for 60 min. A Leica TCS SP8 confocal microscope (Leica, Mannheim, Germany) was used for observations. Pixel quantification was carried out with Image J 1.8.0 software.

### 4.14. RNA-Sequencing Analysis

Total RNA was extracted from control and knockdown C1orf131 groups in A549 cells using the Trizol method, respectively. cDNA library construction and RNA-sequencing analysis (RNA-seq) in this study were performed by Novogene Biotech Co., Ltd. (Beijing, China). GO and KEGG pathway enrichment analysis was then performed on the differentially expressed genes.

### 4.15. Statistical Analysis

All statistical analyses and graphs were conducted using GraphPad Prism 8.3 software. Student’s *t* test was used for comparisons between two groups, while the one-way ANOVA was utilized for comparisons among multiple groups. The Kaplan–Meier curve is a common approach used to analyse survival. All experimental data are expressed as the mean ± standard deviation (SD). Statistically significant differences are defined as * *p* < 0.05, ** *p* < 0.01, and *** *p* < 0.001.

## Data Availability

The RNA-seq raw data have been uploaded to the NCBI database with the accession number PRJNA1099947. The datasets used and/or analysed during the current study are available from the corresponding author upon reasonable request.

## Supplementary Materials

The following supporting information can be downloaded at: https://www.mdpi.com/article/10.3390/ijms25126381/s1.
